# Announcement

**Published:** 1895-01

**Authors:** 


### Editorial.

### ANNOUNCEMENT.

With this issue, Dr. W. S. Goldsmith, assumes the management of the Editorial Department of the Record.

Communications intended for this department should reach this office not later than the 5th of the month to insure publication. The Record will hereafter be issued promptly on the 15th of each month.

